# Chinese Herbal Medicine Combined with Intravitreal Antivascular Growth Factor Agents in Treatment of Macular Edema Secondary to Retinal Vein Occlusion: A Systematic Review and Meta-Analysis

**DOI:** 10.1155/2022/4823677

**Published:** 2022-12-28

**Authors:** Su Xiaojuan, Li Xiaodong, Fu Zhongmei, Ye Hejiang

**Affiliations:** ^1^Department of Ophthalmology, Chengdu University of Traditional Chinese Medicine, Chengdu 610072, Sichuan, China; ^2^Hospital of Chengdu University of Traditional Chinese Medicine, Chengdu 610075, Sichuan, China

## Abstract

**Background:**

Retinal vein occlusion (RVO) is the second most common retinal vascular disease in the world after diabetic retinopathy. Moreover, macular edema (ME) is the main cause of visual impairment in RVO patients. Intravitreal injection of antivascular endothelial growth factor (VEGF) agents is recommended for RVO-ME. However, repeated injections severely limit their efficacy. Chinese herbal medicine (CHM) is widely used in RVO-ME as adjuvant therapy in China.

**Objective:**

The study aims to evaluate the efficacy and safety of anti-VEGF combined with CHM for RVO-ME and to provide reliable evidence for clinical application.

**Methods:**

Seven databases were searched without language or publication status restrictions. Randomized controlled trials (RCTs) comparing anti-VEGF combined with CHM (anti-VEGF + CHM) versus anti-VEGF in participants with RVO-ME were included in this study. The “risk of bias assessment tool” of the Cochrane Handbook was applied to assess the quality of included trials, and RevMan 5.3 software was used for data analysis.

**Results:**

A total of 10 relevant trials with 743 patients were identified. The results showed that BCVA of the anti-VEGF + CHM group significantly improved at 3 months (*P* < 0.00001), 6 months (*P*=0.008), and 12 months (*P*=0.01), and CMT significantly reduced at 1 month (*P*=0.02), 2 months (*P*=0.0009), 3 months (*P* < 0.05), 6 months (*P* < 0.0001), and 12 months (*P* < 0.00001) compared with the anti-VEGF group alone. At the same time, the anti-VEGF + CHM group has a better performance in reducing the number of injections (*P* < 0.05) and improving the total effective rate (*P* < 0.0001). However, regarding adverse events, there was no statistical difference between the two groups (*P*=0.09).

**Conclusions:**

Our results provide promising evidence that anti-VEGF therapy combined with CHM may be more beneficial to patients than anti-VEGF therapy alone. However, because of the low quality and small sample size of the included studies, more rigorous and larger-scale trials were necessary to validate our results. *Registration Number*. CRD42021270262.

## 1. Introduction

Retinal vein occlusion (RVO) is an obstruction of the normal venous system of the retina and the second most common retinal vascular disease after diabetic retinopathy, which seriously affects the visual function and quality of life of patients [1, 2]. The overall prevalence of RVO is 0.72% and it is estimated that RVO will affect 21 million people in Asia in 2040 [3]. Macular edema (ME) is the most serious complication of RVO, which can lead to irreversible visual impairment [4, 5]. ME occurs in 5%–15% of patients with branch retinal vein occlusion (BRVO) within 1 year of onset. Within 2 years of central retinal vein occlusion (CRVO), ME occurs in approximately 30% of non-ischemic CRVO patients and 75% of ischemic CRVO patients [6, 7]. Vascular endothelial growth factor (VEGF) is a key factor in the pathogenesis of RVO-ME. It increases the permeability of blood vessels by increasing the phosphorylation of tight junction proteins, leading to the destruction of the blood-retinal barrier and vascular leakage, triggering the occurrence of ME. Anti-VEGF has therefore become a major treatment option for RVO-ME with promising results [8–10]. However, anti-VEGF agents have a short half-life and require multiple injections, which increased the safety risks for patients, such as intraocular inflammation, intraocular pressure elevation, and ocular hemorrhage [11]. At the same time, it brings a heavy psychological and economic burden to patients and may even lead to family and social problems. Therefore, the study of complementary therapy based on anti-VEGF may have great clinical and social significance.

CHM has the characteristics of comprehensive effect and low price. In the theory of traditional Chinese medicine (TCM), RVO belongs to the category of “sudden blindness.” RVO-ME is a collateral disease in TCM. The basic pathogenesis is vein stasis, and blood stasis turns into edema. Promoting blood circulation and removing blood stasis, promoting diuresis, and eliminating dampness are the most important therapeutic methods in clinical research [12]. TCM is widely used in complementary therapy of RVO-ME in China, but there is a lack of evaluation and evidence on the efficacy and safety of this add-on therapy.

The purpose of this systematic review is to evaluate the efficacy and safety of anti-VEGF combined with CHM Versus anti-VEGF alone in the treatment of RVO-ME and to provide evidence for clinical practice in this specific field.

## 2. Materials and Methods

### 2.1. Methods

This study followed the preferred reporting items for systematic reviews and meta-analysis (PRISMA) statements checklist [13]. The PRISMA 2020 checklist is provided in Supplementary Material S1. Besides, our systematic review protocol was registered at the international prospective register of systematic reviews (PROSPERO) with the registration number CRD42021270262.

### 2.2. Search Strategy

The following electronic databases were searched: PubMed, Embase, Cochrane Library, China National Knowledge Infrastructure (CNKI), Wanfang Database, VIP information resource integration service platform (cqvip), and China Biology Medicine (SinoMed). The search time was from the inception of the database to February 12, 2022, and the search method of combining subject words and free words was adopted. The following grouped keywords were used as search strategy and modified according to different databases: “retinal vein occlusion,” “RVO,” “BRVO,” “CRVO,” “macular edema,” “macular edema, cystoid,” “central retinal edema,” “cystoid macular dystrophy,” “drugs, Chinese herbal,” “Chinese herbal medicine,” “TCM,” “alternative medicine,” “vascular endothelial growth factors,” “anti-VEGF,” “ranibizumab,” “bevacizumab,” “aflibercept,” and “randomized controlled trial “RCT.” No restriction on language was applied. We also manually retrieved relevant articles and clinical studies to obtain the literature as much as we can. No language and status restriction was set in this review. The detailed search strategies are provided in Supplementary Material S2.

### 2.3. Inclusion Criteria

Inclusion criteria were as follows: (1) subjects: patients with RVO-ME; (2) research type: randomized controlled trial (RCT), regardless of whether the literature is blind or not; (3) interventions: patients in the treatment group received anti-VEGF + CHM (including decoction, pill, capsule, Chinese medicinal preparations, and no restricted dosage form). The control group received the same anti-VEGF agents as the treatment group, with or without a placebo; (4) languages of publication are not restricted; (5) one or some related outcomes, including best-corrected visual acuity (BCVA) and central macular thickness (CMT), number of intravitreal injections, total effective rate, and the incidence of adverse events (AE) were reported in the studies; and (6) study had a minimum follow-up of 6 months.

### 2.4. Exclusion Criteria

Exclusion criteria were as follows: (1) case reports, abstracts, reviews, and reports with incomplete data about the macular thickness or best-corrected visual acuity; (2) combined with other retinal diseases; (3) patients received other TCM therapies except for CHM, including acupuncture, moxibustion, cupping, and tuina; (3) trials without a control group; (4) the original document or complete raw data could not be obtained or the raw data could not be extracted or merged; and (5) duplicate published articles and duplicate data.

### 2.5. Data Extraction

Two authors will independently extract data. Any disagreement will be resolved by discussion until consensus is reached or by consulting a third author. The following data will be extracted: author, published year, country, age, gender and number of participants, duration of RVO, intervention measures, follow-up period, BCVA, CMT, number of intravitreal injections, total effective rate, and AE. Any differences in the assessment process will be resolved through consultation. If such differences are not resolved, a third researcher will join the discussion and make a final decision.

The logarithm of the minimum angle of resolution (LogMAR) data on BCVA was extracted from studies. If studies reported BCVA in other units, the values were transformed to LogMAR as described [14].

### 2.6. Quality Evaluation

Based on the bias risk assessment of the Cochrane collaboration tool [15], the methodological quality of the selected studies was evaluated by XS and XL. The assessed items included: random sequence generation (selection bias), allocation concealment (selection bias), blinding of participants and personnel (performance bias), blinding of outcomes assessment (detection bias), incomplete outcomes data (attrition bias), selective reporting (reporting bias), and other biases. Three levels were used to assess the following methodological quality: “low risk of bias (+),” “high risk of bias (−),” and “unclear risk of bias (?).” Consensus on this issue was researched with the help of a third party when necessary.

### 2.7. Statistical Analyses

Review Manager software (RevMan, Version 5.3; Cochrane Collaboration) was utilized to conduct the data analysis of dichotomous and continuous outcome measures. The risk ratio (RR) was adopted for dichotomous variable data as the index of effect value, and mean difference (MD) was adopted for the continuous variables. All of these were expressed as a 95% confidence interval (CI). The heterogeneity was estimated by the chi-squared test, and it was presented as significant when *I*^2^ statistics > 50% or *P* < 0.05 [16]. The random-effects model was applied for the meta-analysis if significant heterogeneity was identified. Otherwise, the fixed-effects model was applied. Feature analyses were performed based on subgroup analyses of the study to explore the sources of heterogeneity. For the study reporting median and interquartile ranges, mean and standard deviation were estimated using the method described by McGrath et al. [17]. In addition, sensitivity analyses were performed to assess the stability of the meta-analysis results. Publication bias was assessed by funnel plot analysis and statistically investigated by Egger's test. *P* < 0.05 was considered statistically significant, except where otherwise specified.

## 3. Results

### 3.1. Study Identification

**Figure 1:**
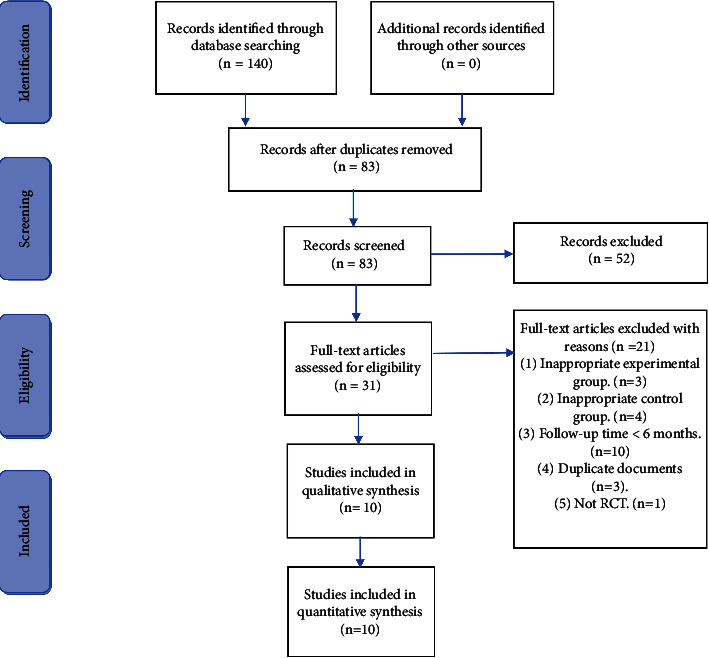
The flow chart of literature screening.

According to the literature retrieval strategies and inclusion and exclusion criteria mentioned previously, 140 articles with possible relevance were retrieved from seven electronic databases. After the removal of 57 duplicates, 83 reports were retrieved. After going through the titles and abstracts, 52 articles were excluded because they failed to meet the inclusion criteria, and the remaining 31 articles were retrieved in full text for further assessment. After further reading, 21 articles were excluded for the reasons listed in Supplementary Material S3. Finally, a total of 10 eligible studies were included in this meta-analysis, with 743 patients in total. The flow chart of the literature screening is shown in Figure 1.

### 3.2. Study Characteristics

The 10 included studies were all conducted in China and published from 2017 to 2021. The sample sizes of the included studies ranged from 42 to 150, with a total of 743 subjects, 376 patients in treatment groups, and 367 patients serving as controls. Among all the studies, two studies were CRVO [18, 19], four studies were BRVO [20–23], and the agents for the remaining studies are unclear. In terms of anti-VEGF agents, five studies used ranibizumab [18, 20, 23–25], two studies used conbercept [21, 22], one study used bevacizumab [19], and the remaining studies did not clarify. Among the primary outcomes, seven studies reported BCVA at different times [19–23, 26, 27], and all studies reported CMT. Among the secondary outcomes, six studies reported the number of intravitreal injections [19–23, 27], and five studies reported the total effective rate [19, 21, 23, 24, 26]. In addition, five studies reported an incidence of overall AE [18, 20–22, 27]. Characteristics of the included studies are presented in Table 1.

### 3.3. Risk of Bias of Included Trials

**Figure 2:**
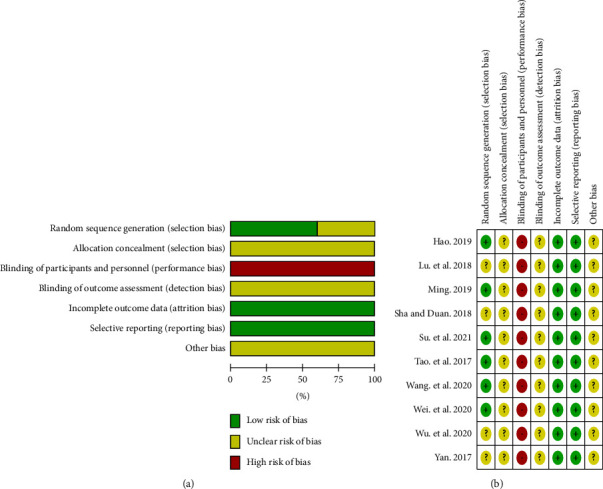
The results of risk of bias assessment.

The results of the risk of bias assessment are shown in (Figures 2(a) and 2(b)). In this systematic review, all 10 trials were reported as RCTs. However, only six studies described their random method [18, 19, 21–23, 26], among which four studies mentioned the “random number table method” explicitly [18, 19, 22, 26], one study was generated by SAS software [21], and one study was mentioned as the “complete random method” [23]. The remaining research only mentions the word “random,” without detailed information about this method. Because no sham injection and placebo were used, the blinding of participants and personnel was assessed as high risk. None of the included studies mentioned the blind method of distribution hiding and result evaluation explicitly. Due to a lack of information, other biases in all studies were also assessed as unclear.

### 3.4. Description of the CHM

The ingredients of CHM in each RCT are listed in Supplementary Material S4. A total of 9 herbal decoctions were used in 10 included studies. The number of herbal components in the formulas varied from 9 to 19. Details of the most commonly used herbs in included studies are listed in Table 2. The most popular form of Chinese herbal medicine is a decoction, which was used in 8/10 studies. All herbal medicines are taken orally. All studies reported the complete ingredients of herbal formulas, among which seven studies mentioned the full dose of related ingredients [18, 20–22, 24–26].

### 3.5. Primary Outcome

#### 3.5.1. BCVA

**Figure 3:**
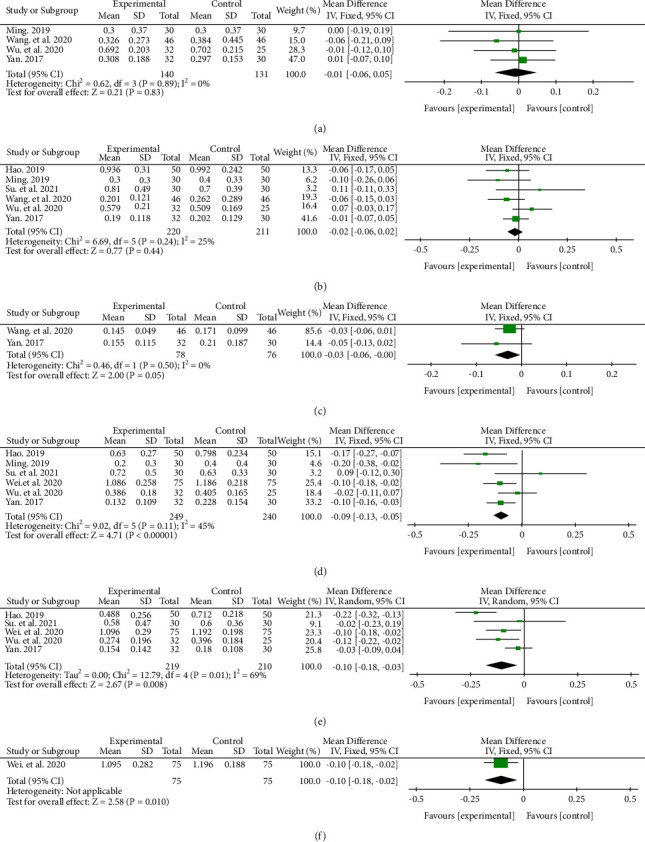
BCVA effects in treatment and control groups at 1 week (a), 1 month (b), 2 months (c), 3 months (d), 6 months (e), and 12 months (f) after treatment.

The result showed that there were no significant differences in BCVA between the two groups at 1 week (MD −0.01, 95% CI (−0.06, 0.05), *P*=0.83), 1 month (MD −0.02, 95% CI (−0.06, 0.02), *P*=0.44), 2 months (MD −0.03, 95% CI (−0.06, −0.00), *P*=0.05), and after treatment in RVO-ME patients. While the anti-VEGF + CHM group had better BCVA at 3 months (MD −0.09, 95% CI (−0.13, −0.05), *P* < 0.00001), 6 months (MD −0.10, 95% CI (−0.18, −0.03), *P*=0.008), and 12 months (MD −0.10, 95% CI (−0.18, −0.02), *P*=0.01) (Figure 3).

The meta-analysis of BCVA at 6 months showed moderate heterogeneity (*P*=0.01, *I*^2^ = 69%), so we conducted a subgroup analysis. Due to the small number of studies, we did not conduct a subgroup analysis based on the types of anti-VEGF agents. In the RVO subtype, the average BCVA improvement between the two groups was significant at the RVO subgroup (*P*=0.0008), while it was not statistically significant at the BRVO subgroup (*P*=0.22) and the CRVO subgroup (*P*=0.85). Meanwhile, in the course of treatment, the average BCVA improvement was significant at ≥12 W subgroup (*P*=0.004), and it was not significant at <12 W subgroup (*P*=0.22). (Supplementary Materials S5 and S6).

#### 3.5.2. CMT

**Figure 4:**
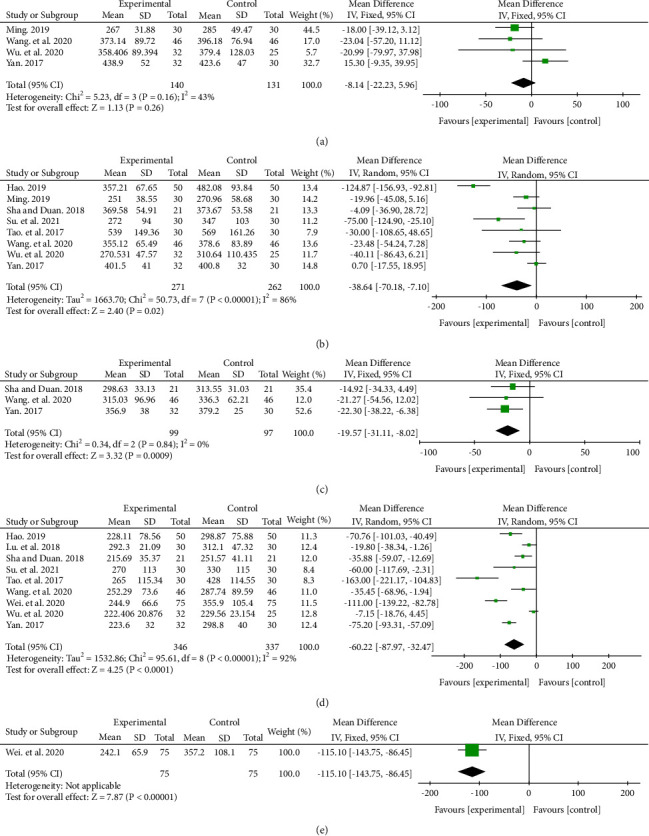
CMT effects in treatment and control groups at 1 week (a), 1 month (b), 2 months (c), 6 months (d), and 12 months (e) after treatment.

Meta-analysis showed that there was no difference between the two groups at 1 week (MD −8.14, 95% CI (−22.23, 5.96), *P*=0.26), but at 1 month (MD −38.64, 95% CI (−70.18, −7.10), *P*=0.02), 2 months (MD −19.57, 95% CI (−31.11, −8.02), *P*=0.0009), 6 months (MD −60.22, 95% CI (−87.97, −32.47), *P* < 0.0001), and 12 months (MD −115.10, 95% CI (−143.75, −86.45 ), *P* < 0.00001), the CMT of anti-VEGF + CHM group was better than that of the anti-VEGF group alone (Figure 4). Due to the high heterogeneity at 3 months, the descriptive analysis was performed. Three studies showed that there was no statistical difference in CMT between the two groups [20, 23, 27]. Five studies showed that the anti-VEGF + CHM group had better CMT than the anti-VEGF group alone, and the difference was statistically significant (*P* < 0.05) [18, 19, 21, 25, 26].

The CMT improvement between the two groups was significant at the CRVO subgroup (*P*=0.004), bevacizumab subgroup (*P*=0.003), and ≥12 W subgroup (*P*=0.008) at 1 month. At the same time, the CMT improvement between the two groups was significant in all 3 subgroups at 6 months, except for the unclear anti-VEGF agents' subgroup (*P*=0.26) (Supplementary Materials S5 and S6).

### 3.6. Secondary Outcomes

#### 3.6.1. Number of Intravitreal Injections

Six studies reported the number of anti-VEGF injections during the follow-up period, with high heterogeneity among studies (*P* < 0.00001, *I*^2^ = 98%) [19–23, 27]. We speculated that the reason for the heterogeneity might be the difference in injection schemes and reinjection standards among studies, so we made a descriptive analysis. One study showed that there was no statistical difference in the number of anti-VEGF injections between the two groups [22]. Five studies showed that the anti-VEGF + CHM group, compared with the anti-VEGF group, could reduce the number of intravitreal injections, and the difference was statistically significant (*P* < 0.05) [19–21, 23, 27].

#### 3.6.2. Total Efficacy Rate

**Figure 5:**
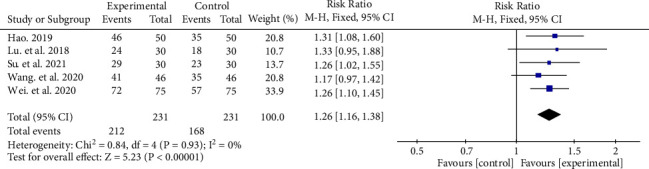
Total effective rate of experimental group and control group.

Five studies reported the total effective rate during the follow-up period [19, 21, 23, 24, 26]. Due to the low statistical heterogeneity (*P*=0.93, *I*^2^ = 0%), a fixed-effect model was used for the meta-analysis. The total effective rate of the anti-VEGF + CHM group was 91.77% (212 divided by 231), while the anti-VEGF group was 72.73% (168 divided by 231), which indicated that the anti-VEGF + CHM group had a better pharmacological effect (RR 1.26, 95% CI (1.16, 1.38), *P* < 0.00001) (Figure 5).

### 3.7. Adverse Effects

**Figure 6:**
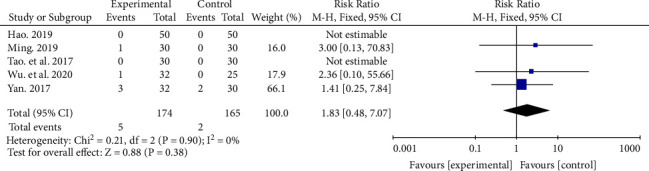
Adverse effects rate of experimental group and control group.

Characteristics of adverse events for included studies are listed in Supplementary Material S7. Five studies recorded adverse events [18, 20–22, 27], including intraocular hypertension and subconjunctival hemorrhage. None of the studies reported serious adverse events (vitreous hemorrhage, retinal hyperplasia, transient cerebral ischemia, and cerebral hemorrhage). Moreover, there was no evidence to show that there was a difference in adverse events between the two groups (RR 1.83, 95% CI (0.48, 7.07), *P*=0.38), and there was no heterogeneity between the two groups (*P*=0.9, *I*^2^ = 0%) (Figure 6).

### 3.8. Sensitivity Analysis

In addition, we performed a sensitivity analysis. Generally, when one particular study was deleted, the remaining studies were reanalysis to see if omitted studies affected the overall estimate significantly. As shown in Supplementary Material S8, sensitivity analysis indicated that Hao's study [21] was the main source of CMT heterogeneity at 1 month. Heterogeneity was reduced after the removal of this study (*P*=0.1, *I*^2^ = 43%) (Supplementary Material S9). Meta-analysis showed a better CMT in the anti-VEGF + CHM group (MD −20.00, 95% CI (−36.89, −3.11), *P*=0.02). There were no specific tests that affected BCVA and CMT performance significantly at 6 months. We speculate that it may be due to the small sample size, which makes it impossible to predict heterogeneity accurately.

### 3.9. Publication Bias

We performed a publication bias test on all studies that reported CMT at 6 months, and Egger's test results showed that there was no risk of publication bias (*P*=0.774 > 0.05).

### 3.10. GRADE Evidence Profile

An overview of the level of evidence is presented in Table 3. Due to the significant ROB and heterogeneity in the study methods, the overall quality of evidence was assessed as very low quality and low quality, which indicated that these estimates were uncertain and that further studies may affect our confidence in estimating the effects of CHM.

## 4. Discussion

We conducted an extensive literature search and identified 10 studies (743 patients) for analysis. The results showed that the overall quality of the evidence was low. Compared with anti-VEGF therapy alone, the statistical results showed that anti-VEGF agents combined with CHM had a potentially positive effect on RVO-ME in improving vision, promoting the resolution of macular edema, and reducing the number of intravitreal injections. In terms of safety, there was no significant difference between the anti-VEGF + CHM group and the anti-VEGF group alone, which indicated that CHM can be safely used in RVO-ME. At present, the evidence showed that CHM is effective and safe for RVO-ME, but it needs to be verified by a more rigorous design of large-scale clinical trials.

Ischemia and hypoxia in the retina will promote the synthesis of VEGF, induce neovascularization and increase vascular permeability, which plays an important role in promoting the generation and progression of RVO and ME. Anti-VEGF is the preferred treatment option for RVO-ME at this stage [28]. However, due to the economic burden caused by repeated injections, patients with RVO-ME are more willing to use CHM alone or in combination with anti-VEGF agents. In their opinion, CHM is effective in improving systemic symptoms, improving visual function, and reducing the number of injections of anti-VEGF. In addition, CHM has been used for thousands of years, and it seems to be relatively safe. However, the efficacy and safety of CHM for RVO-ME are still inconclusive. To the best of our knowledge, this is the first systematic review and meta-analysis of published RCTs to summarize the efficacy and safety of CHM combined with anti-VEGF in the treatment of RVO-ME and provide patients, decision-makers, and clinicians with the latest updated level of evidence.

The eight most commonly used herbs include the following: Chinese angelica root (Radix Angelicae Sinensis), Radix Rehmanniae (*Rehmannia glutinosa*), Safflower flower (Flos Carthami Tinctorii), Poria (Scierotium Poriae Cocos), Szechuan lovage root (Rhizoma Ligustici Chuanxiong), Peach kernel (Prunus persica), Alisma (Rhizoma Alismatis), and red Peony root (*Paeonia veitchii* Lynch). These herbs achieve the effects of nourishing and harmonizing blood, removing blood stasis. It is considered that the basic pathogenesis of RVO-ME in TCM is blood stasis in the eye context. Based on the basic pathogenesis, TCM focuses on the overall regulation and syndrome differentiation and has the characteristics of giving consideration to both the symptoms and the root causes and flexible medication. Pharmacological studies have demonstrated the mechanisms of some drugs in treating RVO-ME. For example, Z-Ligustilide in Chinese angelica root is involved in regulating the secretion of VEGF in ischemic/hypoxic retinopathy and protecting the function and morphology of the retina [29]. Radix Rehmanniae can dilate blood vessels, reduce the permeability of capillary, and inhibit inflammation of vascular endothelium [30]. Quercetin, luteolin, baicalein, and *β*-sitosterol in safflower flower have such biological activities as anti-inflammation, oxidation resistance, and regulation of immunity, which are the material basis for the treatment of RVO [31]. The ethanol extract of Poria can inhibit the nuclear factor-kappa B signaling pathway induced by lipopolysaccharide in RAW 264.7 macrophages, thus reducing the secretion of inflammatory mediators TNF-*α* and IL-1*β*. Poria targets the response of macrophages via the inactivation of the NF-*κ*B signaling pathway [32].

Our findings are consistent with previously published systematic reviews and meta-analyses. Liu et al. evaluated the efficacy of TCM combined with different western medicine therapies in the treatment of RVO-ME and found that the combined therapy had better efficacy than the western medicine therapy in improving the visual acuity, reducing the CMT, and improving the clinical efficacy [33]. However, most of the included studies have a short observation time. RVO-ME is prone to relapse, so long-termfollow-up and observation are needed to obtain the long-term effect of CHM on RVO-ME. Therefore, our study stipulated that the included study should have a minimum follow-up time of 6 months. At the same time, we took the number of anti-VEGF injections as a secondary outcome, because it was necessary and important for evaluating the economy and safety of CHM.

Our meta-analysis has some limitations. First, the overall quality of the included studies is not high and the methodological quality is poor, which may lead to selection bias, implementation bias, and result measurement bias. Secondly, due to the difference in prescription and herbal dosage, there is considerable clinical heterogeneity among these CHMs. Therefore, the combined analysis can only draw the trend of overall efficacy, but cannot draw positive or negative conclusions, which leads to a certain limitation of the extrapolation of the research results. At the same time, none of the included studies focused on the outer layer structure of optical coherence tomography, which is closely related to visual acuity. Future studies are suggested to take external limiting membrane and ellipsoid zone into account, which is more conducive to the interpretation of the results. In addition, since we only retrieved articles in English and Chinese, articles published in other languages, such as Korean and Japanese (CHM is commonly used in East Asian countries), may not be recognized and included in this review, which may lead to deviations in the results. It is suggested that future research should report the generation of a random distribution sequence and the concealment of a random scheme in detail, as well as the withdrawal and dropping off of participants in the study. The sample size calculation method, as well as the composition and dose of CHM, should also be reported in detail. At the same time, the design and report of the study should follow the CONSORT statement.

## 5. Conclusion

In conclusion, the current data are promising but inconclusive. The combination of CHM and anti-VEGF may be more beneficial to RVO-ME than anti-VEGF alone. However, due to the limitation of sample size and methodological quality, a large sample size and multicentre well-designed clinical study are required.

## Figures and Tables

**Table 1:** Characteristics of the included studies.

Study ID	Region	RVO type	No. of patients (T/C)	No. of eyes (T/C)	Length of follow-up(months)	Age		Gender (M/F)		Intervention		Duration of RVO (T/C)	The course of treatment (weeks)	Outcome
						T	C	T	C	T	C			
Su et al. 2021 [19]	China	CRVO	30/30	30/30	6	58.76 ± 14.52	54.2 ± 9.1	13/17	16/14	1.25 mg IVB + CHM	1.25 mg IVB	3.01 ± 3.23/2.85 ± 2.78 (months)	12	a, b, c, d
Wang et al. 2020 [23]	China	BRVO	46/46	46/46	6	53.73 ± 7.15	51.45 ± 6.43	24/22	20/26	0.5 mg IVR + CHM	0.5 mg IVR	1.78 ± 0.44/1.89 ± 0.35 (months)	12	a, b, c, d
Hao 2019 [21]	China	BRVO	50/50	50/50	6	57.09 ± 14.35	58.83 ± 12.43	27/23	26/24	0.5 mg IVC + CHM	0.5 mg IVC	27.2 ± 12.4/28.6 ± 12.2 (days)	24	a, b, c, d
Wei et al. 2020 [26]	China	RVO	75/75	75/75	12	45.2 ± 3.3	45.3 ± 3.4	40/35	39/36	Anti-VEGF + CHM	Anti-VEGF	0.8 ± 0.2/0.8 ± 0.1 (months)	4	a, b, d
Sha and Duan 2018 [25]	China	RVO	21/21	21/21	6	40∼75	40∼75	12/9	10/11	0.5 mg IVR + CHM	0.5 mg IVR	1∼3 (months)	3∼6	b
Wu et al. 2020 [26]	China	RVO	32/25	32/25	6	54.13 ± 11.542	60.84 ± 8.971	19/13	10/15	Anti-VEGF + CHM	Anti-VEGF	NA	12	a, b, c
Tao et al. 2017 [18]	China	CRVO	30/30	30/30	6	55.17 ± 10.66		34/26		0.5 mg IVR + CHM	0.5 mg IVR	57.3 ± 16.9 (days)	12	b
Yan 2017 [20]	China	BRVO	32/30	32/30	6	56.0 ± 0.91	53.9 ± 1.10	18/14	20/10	0.5 mg IVR + CHM	0.5 mg IVR	2.16 ± 0.37/1.89 ± 0.18 (months)	4	a, b, c
Ming. 2019 [22]	China	BRVO	30/30	30/30	6	54.96 ± 13.70	52.92 ± 19.06	11/16	10/15	0.5 mg IVC + CHM	0.5 mg IVC	NA	12	a, b, c
Lu and Wu 2018 [24]	China	RVO	30/30	30/30	6	61.80 ± 7.26	61.17 ± 7.47	15/15	14/16	0.5 mg IVR + CHM	0.5 mg IVR	1.70 ± 0.63/1.68 ± 0.69	24	a, b, d

Note: T: treatment group, C: control group, NA: not available, M/F: male/female, CHM: Chinese herbal medicine, anti-VEGF: antivascular endothelial growth factor, BCVA: best corrected visual acuity, CMT: central macular thickness, IVB: intravitreal bevacizumab, IVR: intravitreal ranibizumab, and IVC: intravitreal conbercept. Outcomes: a: BCVA, b: CMT, c: number of injections, and d: total clinical efficacy.

**Table 2:** Details of the most commonly used herbs in included studies.

Chinese name	English name	Latin name	Family	Number of studies (%)
Danggui	Chinese Angelica Root	Radix Angelicae Sinensis	Umbelliferae	10 (100)
Sheng Di Huang	Radix Rehmanniae	*Rehmannia glutinosa*	Scrophulariaceae	9 (90)
Honghua	Safflower flower	Flos Carthami Tinctorii	Asteraceae	7 (70)
Fuling	Poria	Scierotium Poriae Cocos	Polyporaceae	7 (70)
Chuanxiong	Szechuan Lovage Root	Rhizoma Ligustici Chuanxiong	Umbelliferae	7 (70)
Taoren	Peach kernel	Prunus persica	Rosaceae	6 (60)
Zexie	Alisma	Rhizoma Alismatis	Alismaceae	5 (50)
Chishao	Red Peony Root	*Paeonia veitchii* Lynch	Ranunculaceae	5 (50)

**Table 3:** GRADE for quality of evidence profile.

Quality assessment							No. of patients		Absolute effect (95% CI)	Quality
No. of studies	Study design	Risk of bias	Inconsistency	Indirectness	Imprecision	Other considerations	CHM combined with anti-VEGF agents	Anti-VEGF agents		
*BCVA 1W*										
4	RCT	Serious^a^	Not serious	Not serious	Serious^d^	None	140	131	MD −0.01 (−0.06, 0.05)	⊕⊕⃝⃝LOW

*BCVA 1M*										
6	RCT	Serious^a^	Serious^c^	Not Serious	Not serious	None	220	211	MD −0.02 (−0.06, 0.02)	⊕⊕⃝⃝LOW

*BCVA 2M*										
2	RCT	Serious^a^	Not serious	Not serious	Serious^d^	None	78	76	MD 0.04 (−0.01, 0.08)	⊕⊕⃝⃝LOW

*BCVA 3M*										
6	RCT	Serious^a,b^	Serious^c^	Not serious	Not serious	None	249	240	MD −0.09 (−0.13,−0.05)	⊕⊕⃝⃝LOW

*BCVA 6M*										
5	RCT	Serious^a,b^	Serious^c^	Not serious	Not serious	None	219	210	MD −0.10 (−0.18,−0.03)	⊕⊕⃝⃝LOW

*BCVA 12M*										
1	RCT	Serious^a^	Not serious	Not serious	Serious^d^	None	75	75	MD −0.10 (−0.18,−0.02)	⊕⊕⃝⃝LOW

*CMT 1W*										
4	RCT	Serious^a^	Not serious	Not serious	Serious^d^	None	140	131	MD −8.14 (−22.23, 5.96)	⊕⊕⃝⃝LOW

*CMT 1M*										
8	RCT	Serious^a^	Serious^c^	Not serious	Serious^d^	None	271	262	MD −38.64 (−70.18, −7.10)	⊕⃝⃝⃝VERY LOW

*CMT 2M*										
3	RCT	Serious^a^	Not serious	Not serious	Serious^d^	None	99	97	MD −19.57 (−31.11, −8.02)	⊕⊕⃝⃝LOW

*CMT 3M*										
8	RCT	Serious^a^	Serious^c^	Not serious	Serious^d^	None	325	316	MD −61.81 (−99.84, −23.77)	⊕⃝⃝⃝VERY LOW

*CMT 6M*										
9	RCT	Serious^a^	Serious^c^	Not serious	Serious^d^	None	346	337	MD −60.22 (−87.97, −32.47)	⊕⃝⃝⃝VERY LOW

*CMT 12M*										
1	RCT	Serious^a^	Not serious	Not serious	Serious^d^	None	75	75	MD −115.10 (−143.75, −86.45)	⊕⊕⃝⃝LOW

*Number of anti-VEGF injections*										
6	RCT	Serious^a^	Serious^c^	Not serious	Not Serious	None	220	211	MD −1.03 (−1.44, −0.61)	⊕⊕⃝⃝LOW

*Total effective rate*										
5	RCT	Serious^a^	Not serious	Not serious	Not serious	None	231	231	RR 1.26 (1.16, 1.38)	⊕⊕⊕⃝MODERATE

^a^missing detail about blindness, allocation concealment, and other biases, ^b^no clear randomization procedure is described, ^c^high heterogeneity leads to serious inconsistency, and ^d^the wide confidence interval indicates imprecision and the sample size is small.

## Data Availability

Registration number: CRD42021270262. The original contributions presented in the study are included in the article/Supplementary Material; further inquiries can be directed to the corresponding author.

## Abbreviations

RVO: Retinal vein occlusion
ME: Macular edema
VEGF: Vascular endothelial growth factor
CNKI: China national knowledge infrastructure
cqvip: VIP information resource integration service platform
SinoMed: China biology medicine
RCT: Randomized controlled trials
CHM: Chinese herbal medicine
BRVO: Branch retinal vein occlusion
CRVO: Central retinal vein occlusion
TCM: Traditional Chinese medicine
PRISMA: Preferred reporting items for systematic reviews and meta-analysis
BCVA: Best-corrected visual acuity
CMT: Central macular thickness
AE: Adverse events
LogMAR: Logarithm of the minimum angle of resolution
RR: Risk ratio
MD: Mean difference
CI: Confidence interval.

## Additional Points

All claims expressed in this article are solely those of the authors and do not necessarily represent those of their affiliated organizations or those of the publisher, the editors, and the reviewers. Any product that may be evaluated in this article or claim that may be made by its manufacturer is not guaranteed or endorsed by the publisher.

## Supplementary Materials

Supplementary Materials Supplementary Material S1: PRISMA 2020 Checklist. Supplementary Material S2: search terms for each database. Supplementary Material S3: a list of excluded studies by reading the full text. Supplementary Material S4: components of Chinese herbal formulas used in the included trials. Supplementary Material S5: table of subgroup analysis. Supplementary Material S6: forest plot of subgroup analysis. Supplementary Material S7: characteristics of adverse events for included studies. Supplementary Material S8: a sensitivity analysis. Supplementary Material S9: forest plot of CMT at 1 month (after removing Hao's study).
