# Supplementary material for: Chinese Herbal Medicine Combined with Intravitreal Antivascular Growth Factor Agents in Treatment of Macular Edema Secondary to Retinal Vein Occlusion: A Systematic Review and Meta-Analysis
Source: J Ophthalmol. 2022 Dec 28;2022:4823677. doi: 10.1155/2022/4823677 (PMC11390223; doi:10.1155/2022/4823677)
Supplement: Supplementary Materials — Supplementary Material S1: PRISMA 2020 Checklist. Supplementary Material S2: search terms for each database. Supplementary Material S3: a list of excluded studies by reading the full text. Supplementary Material S4: components of Chinese herbal formulas used in the included trials. Supplementary Material S5: table of subgroup analysis. Supplementary Material S6: forest plot of subgroup analysis. Supplementary Material S7: characteristics of adverse events for included studies. Supplementary Material S8: a sensitivity analysis. Supplementary Material S9: forest plot of CMT at 1 month (after removing Hao's study). [file 4823677.f1.zip › Supplementary Material S2.pdf]

Supplementary Material S2. Search terms for each database.

Databases	Search items
Cochrane Library	#1 MeSH descriptor: [Retinal Vein Occlusion] explode all trees<br>#2 (retina*):ti,ab,kw (Word variations have been searched)<br>#3 (ve?n*):ti,ab,kw OR (vessel*):ti,ab,kw OR (vascula*):ti,ab,kw (Word variations have been searched)<br>#4 (occlu*):ti,ab,kw OR (obstruct*):ti,ab,kw OR (block*):ti,ab,kw OR (thromb*):ti,ab,kw OR (embol*):ti,ab,kw (Word variations have been searched)<br>#5 (clot*):ti,ab,kw (Word variations have been searched)<br>#6 #4 or #5<br>#7 #2 and #3 and #6<br>#8 (RVO):ti,ab,kw OR (CRVO):ti,ab,kw OR (BRVO):ti,ab,kw (Word variations have been searched)<br>#9 #1 or #7 or #8<br>#10 MeSH descriptor: [Macular Edema] explode all trees<br>#11 (Cystoid Macular Edema, Postoperative):ti,ab,kw OR (Macular Edema, Cystoid):ti,ab,kw OR (Edema, Cystoid Macular):ti,ab,kw OR (Cystoid Macular Dystrophy):ti,ab,kw OR (Macular Dystrophy, Dominant Cystoid):ti,ab,kw (Word variations have been searched)<br>#12 (Central Retinal Edema, Cystoid):ti,ab,kw OR (Cystoid Macular Edema):ti,ab,kw (Word variations have been searched)<br>#13 #11 or #12<br>#14 #10 or #13<br>#15 MeSH descriptor: [Drugs, Chinese Herbal] explode all trees<br>#16 (TCM):ti,ab,kw OR (Chinese medicine):ti,ab,kw OR (Chinese traditional):ti,ab,kw (Word variations have been searched)<br>#17 (herb*):ti,ab,kw OR (alternative medicine):ti,ab,kw OR (Chinese patent medicine):ti,ab,kw OR (decoction):ti,ab,kw OR (prescription):ti,ab,kw (Word variations have been searched)<br>#18 #16 or #17<br>#19 #15 or #18<br>#20 #9 and #14 and #19

Pubmed	#1 "Retinal Vein Occlusion"[Mesh]<br>#2 "retina*"[Tiab]<br>#3 "ve?n*"[Tiab] OR "vessel*"[Tiab] OR "vascula*"[Tiab]<br>#4 "occlu*"[Tiab] OR "obstruct*"[Tiab] OR "block*"[Tiab] OR "thromb*"[Tiab] OR "embol*"[Tiab] OR "clot*"[Tiab]<br>#5 #2 AND #3 AND #4<br>#6 "RVO"[Tiab] OR "BRVO"[Tiab] OR "CRVO"[Tiab]<br>#7 #1 OR #5 OR #6<br>#8 "Macular Edema"[Mesh]<br>#9 "edema macular"[Tiab] OR "cystoid macular edema postoperative"[Tiab] OR "macular edema cystoid"[Tiab] OR "edema cystoid macular"[Tiab] OR "cystoid macular dystrophy"[Tiab]<br>#10 #8 OR #9<br>#11 "drugs, chinese herbal"[Mesh]<br>#12 "TCM"[Tiab] OR "chinese medicine"[Tiab] OR "chinese traditional"[Tiab] OR "herb*"[Tiab] "alternative medicine"[Tiab] OR "chinese patent medicine"[Tiab] OR "decoction"[Tiab] OR "prescription"[Tiab]<br>#13 #11 OR #12<br>#14 "randomized controlled trial" [pt] OR "controlled clinical trial" [pt] OR "randomized" [tiab] OR "placebo" [tiab] OR "drug therapy" [sh] OR "randomly" [tiab] OR "trial" [tiab] OR "groups" [tiab]<br>#15 #7 AND #10 AND #13 AND #14
EMBASE	#1 'retina vein occlusion'/exp<br>#2 retina*:ti,ab,kw AND (ve?n*:ti,ab,kw OR vessel*:ti,ab,kw OR vascula*:ti,ab,kw) AND (occlu*:ti,ab,kw OR obstruct*:ti,ab,kw OR block*:ti,ab,kw OR thromb*:ti,ab,kw OR embol*:ti,ab,kw OR clot*:ti,ab,kw)<br>#3 rvo:ti,ab,kw OR brvo:ti,ab,kw OR crvo:ti,ab,kw<br>#4 #1 OR #2 OR #3<br>#5 'macular edema'/exp<br>#6 'cystoid macular edema, postoperative':ti,ab,kw OR 'macular edema, cystoid':ti,ab,kw OR 'edema, cystoid macular':ti,ab,kw OR 'cystoid macular dystrophy':ti,ab,kw OR 'macular dystrophy, dominant cystoid':ti,ab,kw OR 'central retinal edema, cystoid':ti,ab,kw OR

	'cystoid macular edema':ti,ab,kw<br>#7 #5 OR #6<br>#8 'chinese drug'/exp<br>#9 tcm:ti,ab,kw OR 'chinese medicine':ti,ab,kw OR 'chinese traditional':ti,ab,kw OR herb*:ti,ab,kw OR 'alternative medicine':ti,ab,kw OR 'chinese patent medicine':ti,ab,kw OR decoction:ti,ab,kw OR prescription:ti,ab,kw<br>#10 #8 OR #9<br>#11 'crossover procedure':de OR 'double-blind procedure':de OR 'randomized controlled trial':de OR 'single-blind procedure':de OR random*:de,ab,ti OR factorial*:de,ab,ti OR crossover*:de,ab,ti OR ((cross NEXT/1 over*):de,ab,ti) OR placebo*:de,ab,ti OR ((doubl* NEAR/1 blind*):de,ab,ti) OR ((singl* NEAR/1 blind*):de,ab,ti) OR assign*:de,ab,ti OR allocat*:de,ab,ti OR volunteer*:de,ab,ti<br>#12 #4 AND #7 AND #10 AND #11
CNKI	主题=(视网膜静脉阻塞 + 视网膜中央静脉阻塞 + 视网膜分支静脉阻塞) AND (主题=黄斑水肿) AND (主题=中药 + 中医 + 中西医 + 成药 + 草药 + 注射剂 + 汤 + 方 + 片 + 丸) AND (主题=抗 VEGF + 雷珠单抗 + 康柏西普 + 阿柏西普 + 贝伐单抗)
Sinomed	("视网膜静脉阻塞"[常用字段:智能] OR "视网膜中央静脉阻塞"[常用字段:智能] OR "视网膜分支静脉阻塞"[常用字段:智能]) AND "黄斑水肿"[常用字段:智能] AND （"中药"[常用字段:智能] OR "中医"[常用字段:智能] OR "中西医"[常用字段:智能] OR "成药"[常用字段:智能] OR "草药"[常用字段:智能] OR "注射剂"[常用字段:智能] OR "汤"[常用字段:智能] OR "方"[常用字段:智能] OR "片"[常用字段:智能] OR "丸" [常用字段:智能]）AND （"抗 VEGF"[常用字段:智能] OR "雷珠单抗"[常用字段:智能] OR "康柏西普"[常用字段:智能] OR "阿柏西普"[常用字段:智能] OR "贝伐单抗"）
VIP	(M=(视网膜静脉阻塞 OR 视网膜中央静脉阻塞 OR 视网膜分支静脉阻塞)AND M=(黄斑水肿)) AND (M=(中药 OR 中医 OR 中西医 OR 成药 OR 草药 OR 注射剂 OR 汤 OR 方 OR 片 OR 丸) OR R=(中药 OR 中医 OR 中西医 OR 成药 OR 草药 OR 注射剂 OR 汤 OR 方 OR 片 OR 丸)) AND (M=(抗 VEGF OR 雷珠单抗 OR 康柏西普 OR 阿柏西普 OR 贝伐单抗) OR R=(抗 VEGF OR 雷珠单抗 OR 康柏西普 OR 阿柏西普 OR 贝伐单抗))
Wanfang Data	主题:(视网膜静脉阻塞 OR 视网膜中央静脉阻塞 OR 视网膜分支静脉阻塞) AND 主题:(“黄斑水肿”) AND 主题:(中药 OR 中医 OR 中西医 OR 成药 OR 草药 OR 注射剂 OR 汤 OR 方 OR 片 OR 丸) AND 主题:(抗 VEGF OR 雷珠单抗 OR 康柏西普 OR 阿柏西普 OR 贝伐单抗)
