# Supplementary material for: Chinese Herbal Medicine Combined with Intravitreal Antivascular Growth Factor Agents in Treatment of Macular Edema Secondary to Retinal Vein Occlusion: A Systematic Review and Meta-Analysis
Source: J Ophthalmol. 2022 Dec 28;2022:4823677. doi: 10.1155/2022/4823677 (PMC11390223; doi:10.1155/2022/4823677)
Supplement: Supplementary Materials — Supplementary Material S1: PRISMA 2020 Checklist. Supplementary Material S2: search terms for each database. Supplementary Material S3: a list of excluded studies by reading the full text. Supplementary Material S4: components of Chinese herbal formulas used in the included trials. Supplementary Material S5: table of subgroup analysis. Supplementary Material S6: forest plot of subgroup analysis. Supplementary Material S7: characteristics of adverse events for included studies. Supplementary Material S8: a sensitivity analysis. Supplementary Material S9: forest plot of CMT at 1 month (after removing Hao's study). [file 4823677.f1.zip › Supplementary Material S3.pdf]

Supplementary Material S3. A list of excluded studies by reading full text.

Inappropriate experimental group. (n=3)

1. Luo, J., Wang, S., Zou, R., He, F., and Liao, H. (2020). Clinical study of Huoxue Lishui Mingmu Decoction in treating macular edema secondary to retinal vein occlusion. Chinese medicine modern distance education of China 18(14), 64-66.
2. Que, J., and Li, D. (2019). Clinical observation on the consolidation therapeutic effect of Maizhiling combined with Fuming tablet on RVO-ME anti-VEGF treatment in the later stage. World latest medicine information 19, 137-139.
3. Wang, H., and Wei, Q. (2017). Observation on the clinical effect of treating central retinal vein occlusion with macular edema from the conclusion of phlegm and blood stasis. Tianjin Journal of Traditional Chinese Medicine 34, 305-308.

Inappropriate control group. (n=4)

1. Duan, L. (2019). Clinical analysis of Compaxip injection in the treatment of retinal vein occlusion complicated with macular edema. World latest medicine information 19, 134-143.
2. Fan, Z., and Huang, H. (2020). Effects of different treatments on macular edema caused by branch retinal vein occlusion. Health care guide 46.
3. Tao, R., Chen, M., and Jing, W. (2016). Clinical observation on treatment of macular edema with traditional Chinese medicine for invigorating qi, promoting blood circulation and promoting diuresis combined with laser photocoagulation. Chinese Modern Doctor 54(21), 79-82.
4. Yang, S. (2017). Comparison of curative effects of traditional Chinese medicine combined with different anti-VEGF drugs injected into vitreous cavity on branch retinal vein occlusion with macular edema. Journal of Qiqihar Medical University 38(22), 2663-2664.

Follow-up time < 6 months. (n=10)

1. Du, H., Rollin, W., Li, J., Luo, H., Jian, Y., and Cai, J. (2020). Clinical study of Huoxue Mingmu Decoction combined with ranibizumab in treating macular edema secondary to non-ischemic branch retinal vein occlusion. Journal of Emergency in Traditional Chinese Medicine 29(08), 1381-1383.
2. Gao, L. (2020). Clinical observation on the treatment of macular edema caused by central retinal vein occlusion with traditional Chinese medicine for promoting blood circulation and inducing diuresis combined with Compaxip. master's thesis, Guangxi University of Traditional Chinese Medicine.
3. Ke, F., Chen, S., Hu, Y., and Li, S. (2020). Effect of Xuefu Zhuyu Decoction combined with Leizhumab injection on macular edema secondary to branch retinal vein occlusion. Chinese Journal of Clinical Rational Drug Use 13(29), 101-103.
4. Ke, X., and Lin, Y. (2016). Modified Buyanghuanwu Decoction combined with Compaxip in treating 14 cases of macular edema due to retinal vein occlusion of qi deficiency and blood stasis. Fujian Journal of Traditional Chinese Medicine (3), 42-43.
5. Tan, M., and Xiao, Z. (2021). The curative effect of Tongmai Jingming Decoction combined with Compaxip intravitreal injection on macular edema secondary to

retinal vein occlusion and its influence on VEGF, bFGF and SDF-1 in aqueous humor. Chinese Journal of Pharmacy 30(04), 230-234.
6. Wang, H., and Jiang, Y. (2020). Effect of Xuefu Zhuyu Decoction combined with Compaxip on the level of vision and coagulation function in patients with macular edema due to retinal vein occlusion. Modern Medicine and Health Research Electronic Journal 4(13), 72-74.
7. Wu, Y., and Chen, G. (2019). Treatment of 31 cases of retinal vein occlusion complicated with macular edema by intravitreal injection of traditional Chinese medicine combined with ranibizumab. Zhejiang Journal of Traditional Chinese Medicine (5), 343.
8. Xie, Y., Shi, L., and Yuan, Y. (2021). Analysis of therapeutic effect of Jiawei Taohong Siwu Decoction combined with intravitreal injection of Compaxip on macular edema secondary to BRVO. Guizhou Medical Journal 45(04), 577-578.
9. Yan, H., Wu, S., Fei, Y., and Wang, D. (2020). To observe the therapeutic effect of Taohong Siwu Decoction combined with ranibizumab on macular edema caused by retinal vein occlusion. Journal of Frontier of Medicine 10(6), 208-209.
10.Zhuang, J. (2021). Study on the clinical efficacy and safety of Hexue Mingmu Tablet combined with ranibizumab in the treatment of retinal vein occlusion with macular edema. Chinese Archives of Traditional Chinese Medicine 39(07), 172-175.

Duplicate documents (n=3).

1. Hao, X., Xie, L., Xu, J., Li, X., Jin, Q., Sun, M., et al. (2021). Observation on therapeutic effect of Quji Tongluo recipe combined with anti-VEGF drugs on macular microvascular injury repair. China Journal of Chinese Ophthalmology 31(02), 94-99.
2. Hao, X., Xie, L., Xu, J., Li, X., Lu, B., and Sun, M. (2020). Clinical study on the treatment of macular edema caused by non-ischemic branch retinal vein occlusion with Quji Tongluo recipe combined with anti-VEGF. China Journal of Chinese Ophthalmology 30(04), 244-249.
3. Wu, H. (2018). Clinical observation of traditional Chinese medicine combined with anti-vascular endothelial growth factor drugs in the treatment of macular edema caused by retinal vein occlusion. master's thesis, Nanjing University of Traditional Chinese Medicine.

Not RCT. (n=1)

1. Liu, Z. (2021). Study on the application of traditional Chinese medicine combined with anti-VEGF in the treatment of macular edema caused by retinal vein occlusion. master's thesis, Beijing University of Traditional Chinese Medicine.
