# Supplementary material for: Chinese Herbal Medicine Combined with Intravitreal Antivascular Growth Factor Agents in Treatment of Macular Edema Secondary to Retinal Vein Occlusion: A Systematic Review and Meta-Analysis
Source: J Ophthalmol. 2022 Dec 28;2022:4823677. doi: 10.1155/2022/4823677 (PMC11390223; doi:10.1155/2022/4823677)
Supplement: Supplementary Materials — Supplementary Material S1: PRISMA 2020 Checklist. Supplementary Material S2: search terms for each database. Supplementary Material S3: a list of excluded studies by reading the full text. Supplementary Material S4: components of Chinese herbal formulas used in the included trials. Supplementary Material S5: table of subgroup analysis. Supplementary Material S6: forest plot of subgroup analysis. Supplementary Material S7: characteristics of adverse events for included studies. Supplementary Material S8: a sensitivity analysis. Supplementary Material S9: forest plot of CMT at 1 month (after removing Hao's study). [file 4823677.f1.zip › Supplementary Material S4.pdf]

Supplementary Material S4. Components of Chinese herbal formulas used in the included trials.

Study	Chinese herbal medicine	Components	Dosage formulation
Su. et al. 2021	Hexuemingmu Tablet	Cattail pollen (Puhuang, Typha angustifolia L.), Salvia Root (Danshen, Radix Salviae Miltiorrhizae), Radix Rehmanniae (Dihuang, Rehmannia glutinosa (Gaert.) Libosch.), Herba Ecliptea (Mohanlian, Herba Ecliptae Eclipta prostrala L.), Chrysanthemum (Juhua, Dendranthema morifolium (Ramat.) Tzvel.), Baical Skullcap Root (Huangqin, Radix Scutellariae Baicalensis), Cassia Seed (Juemingzi,Catsia tora Linn), Plantain Seed (Cheqianzi, Semen Plantaginis), Motherwort Fruit (Chongweizi, Leonurus japonicus Houtt.), Glossy Privet Fruit (Nvzhenzi, Fructus Ligustri Lucidi), Prunellae Spica (Xiakucao, Prunella vulgaris Linn.), radix gentianae (Longdan, Gentiana scabra Bunge), Radix curcumae (Yujin, Curcuma aromatica Salisb.), Scouring Rush (Muzei, Equisetum hyemale Linn.), Red Peony Root (Chishao, Paeonia veitchii Lynch), Tree Peony Bark (Mudanpi,Paeonia suffruticosaAndr.), Hawthorn Fruit (Shanzha, Crataegus pinnatifida Bge.), Chinese Angelica Root (Danggui, Radix Angelicae Sinensis), Szechuan Lovage Root (Chuanxiong, Rhizoma Ligustici Chuanxiong)	Tablet
Wang. et al. 2020	Huayu Lishui Decoction	Peach kernel (Taoren, Prunus persica(L.)Batsch), Safflower Flower (Honghua, Flos Carthami Tinctorii), Poria (Fuling, Scierotium Poriae Cocos), Red Peony Root (Chishao, Paeonia veitchii Lynch), Szechuan Lovage Root (Chuanxiong, Rhizoma Ligustici Chuanxiong), Cassia twig (Guizhi, Ramulus Cinnamomi Cassiae), Radix Rehmanniae (Dihuang,Rehmannia glutinosa (Gaert.) Libosch.), Chinese Angelica Root (Danggui, Radix Angelicae Sinensis), Root of the Balloon Flower (Jiegeng, Platycodi Radix), Bittet Orange (Zhiqiao, Fructus Aurantii), White Atractylodes Rhizome (Baizhu, Rhizoma Atractylodis Macrocephalae), Radix Glycyrrhizae Preparata (Zhigancao, Glycyrrhiza uralensis Fisch.)	Decoction
Hao. 2019	Quji Tongluo Decoction	Peach kernel (Taoren, Prunus persica(L.)Batsch) 10g, Safflower Flower (Honghua, Flos Carthami Tinctorii)5g, Radix Rehmanniae (Dihuang,Rehmannia glutinosa (Gaert.) Libosch.) 20g, Chinese Angelica Root (Danggui, Radix Angelicae Sinensis) 10g, Membrane of Chicken Gizzard (Jineijin,Endothelium Corneum Gigeriae galli) 10g, Rhizoma Pinelliae Praeparatum (Fabanxia, Arisaema erubescens (Wall.) Schott) 10g, Tangerine Peel (Chenpi, Pericarpium Citri Reticulatae) 8g, Poria (Fuling, Scierotium Poriae Cocos) 15g, Sanchi (sanqi, Panax notoginseng) 3g, Ledebouriella Root (Fangfeng, Radix Ledebouriellae Divaricatae) 5g.	Decoction

Wei. et al. 2020	Xuefu Zhuyu Decoction	Peach kernel (Taoren, Prunus persica(L.)Batsch) 12g, Safflower Flower (Honghua, Flos Carthami Tinctorii) 9g, Chinese Angelica Root (Danggui, Radix Angelicae Sinensis)9 g, Radix Rehmanniae (Dihuang, Rehmannia glutinosa (Gaert.) Libosch.) 9g, Root of bidentate achyranthes (Niuxi, Achyranthes bidentata Blume) 9g, Szechuan Lovage Root (Chuanxiong, Rhizoma Ligustici Chuanxiong) 6g, Root of the Balloon Flower (Jiegeng, Platycodi Radix) 6g, Red Peony Root (Chishao, Paeonia veitchii Lynch) 6g, Bittet Orange (Zhiqiao, Fructus Aurantii) 6g,Radix Glycyrrhizae Preparata (Zhigancao, Glycyrrhiza uralensis Fisch.) 6g, Thorowax Root (Chaihu, Radix Bupleuri) 6g, Alisma (Zexie, Rhizoma Alismatis) 8g.	Decoction
Sha and Duan. 2018	CHM	Peach kernel (Taoren, Prunus persica(L.)Batsch) 12g, Safflower Flower (Honghua, Flos Carthami Tinctorii) 12g, Salvia Root (Danshen, Radix Salviae Miltiorrhizae) 12g, Szechuan Lovage Root (Chuanxiong, Rhizoma Ligustici Chuanxiong) 12g, Chinese Angelica Root (Danggui, Radix Angelicae Sinensis)12g, Radix Rehmanniae (Dihuang, Rehmannia glutinosa (Gaert.) Libosch.)12g, Thorowax Root (Chaihu, Radix Bupleuri) 12g, Red Peony Root (Chishao, Paeonia veitchii Lynch)12g, Root of Pilose Asiabell (Dangshen, Codonopsis pilosula (Franch.) Nannf.) 20g, Poria (Fuling, Scierotium Poriae Cocos) 15g, White Atractylodes Rhizome (Baizhu, Rhizoma Atractylodis Macrocephalae) 15g, Alisma (Zexie, Rhizoma Alismatis) 12g, Radix Glycyrrhizae Preparata (Zhigancao, Glycyrrhiza uralensis Fisch.) 6g.	Decoction
Wu. et al. 2020	Taohong Siwu Decoction	Safflower Flower (Honghua, Flos Carthami Tinctorii), Chinese Angelica Root (Danggui, Radix Angelicae Sinensis), White Peony Root (Baishaoyao, Radix Albus Paeoniae Lactiflorae), Szechuan Lovage Root (Chuanxiong, Rhizoma Ligustici Chuanxiong), Radix Rehmanniae(Dihuang,Rehmannia glutinosa (Gaert.) Libosch.), Alisma (Zexie, Rhizoma Alismatis), Poria (Fuling, Scierotium Poriae Cocos), Coix seed (Yiyiren, Semen Coicis), Pseudostellaria Root (Taizishen, Pseudostellariae Radix)	Decoction
Tao. et al. 2017	Taohong Siwu Decoctionand Siling Powder	Salvia Root (Danshen, Radix Salviae Miltiorrhizae) 30g, Sanchi (Sanqi, Panax notoginseng)10g, Chinese Angelica Root (Danggui, Radix Angelicae Sinensis)15g, Szechuan Lovage Root (Chuanxiong, Rhizoma Ligustici Chuanxiong)10g, Red Peony Root (Chishao, Paeonia veitchii Lynch)15g, Radix Rehmanniae (Dihuang,Rehmannia glutinosa (Gaert.) Libosch.)15g, Poria (Fuling, Scierotium Poriae Cocos) 30g, Polyporus Sclerotium (Zhuling, Sclerotium Polypori Umbellati) 15g, Alisma (Zexie, Rhizoma Alismatis) 30g, White Atractylodes Rhizome (Baizhu, Rhizoma Atractylodis Macrocephalae) 15g, Cassia twig (Guizhi, Ramulus Cinnamomi Cassiae) 10g, Plantain Seed (Cheqianzi, Semen Plantaginis) 30g, Motherwort Fruit (Chongweizi, Leonurus japonicus Houtt.) 20g	Decoction

Yan. 2017	Jiawei Siwu Decoction and Wuling Powder	Peach kernel (Taoren, Prunus persica(L.)Batsch) 10g, Safflower Flower (Honghua, Flos Carthami Tinctorii) 10g, Chinese Angelica Root (Danggui, Radix Angelicae Sinensis) 12g, Radix Rehmanniae (Dihuang, Rehmannia glutinosa (Gaert.) Libosch.) 12g, Poria (Fuling, Scierotium Poriae Cocos) 20g, White Atractylodes Rhizome (Baizhu, Rhizoma Atractylodis Macrocephalae) 15g, Polyporus Sclerotium (Zhuling, Sclerotium Polypori Umbellati) 15g, Alisma (Zexie, Rhizoma Alismatis), Plantain Seed (Cheqianzi, Semen Plantaginis) 15g, Radix Glycyrrhizae Preparata (Zhigancao, Glycyrrhiza uralensis Fisch.) 5g.	Decoction
Ming. 2019	Quji Tongluo Decoction	Peach kernel (Taoren, Prunus persica(L.)Batsch) 10g, Safflower Flower (Honghua, Flos Carthami Tinctorii) 5g, Radix Rehmanniae (Dihuang, Rehmannia glutinosa (Gaert.) Libosch.)20g, Chinese Angelica Root (Danggui, Radix Angelicae Sinensis)10g, Membrane of Chicken Gizzard (jineijin, Endothelium Corneum Gigeriae galli)10g, Rhizoma Pinelliae Praeparatum (Fabanxia, Arisaema erubescens (Wall.) Schott) 10g, Tangerine Peel (Chenpi, Pericarpium Citri Reticulatae) 8g, Poria (Fuling, Scierotium Poriae Cocos) 15g, Sanchi (Sanqi, Panax notoginseng) 3g, Ledebouriella Root (Fangfeng, Radix Ledebouriellae Divaricatae) 5g.	Decoction
Lu. et al. 2018	Lingqi Huangban Granule	Chinese Angelica Root (Danggui, Radix Angelicae Sinensis) 10g, Szechuan Lovage Root (Chuanxiong, Rhizoma Ligustici Chuanxiong) 10g, Semen Cuscutea (Tusizi, Cuscuta chinensis Lam.) 10g, Lucid Ganoderma (Lingzhi, Ganoderma)10g, Rhizoma Atractylodis (Cangzhu, Atractylodis Rhizoma) 10g, Salvia Root (Danshen, Radix Salviae Miltiorrhizae) 10g, Root of Pilose Asiabell (Dangshen, Codonopsis pilosula (Franch.) Nannf.) 10g, Seaweed (Haizao，Sargassum) 10g, Desertliving Cistanche Herb (Roucongrong, Cistanches Herba)10g, Babury Wolfberry Fruit (Gouqizi, Lycii Fructus) 10g.	Granule
