# Supplementary material for: Chinese Herbal Medicine Combined with Intravitreal Antivascular Growth Factor Agents in Treatment of Macular Edema Secondary to Retinal Vein Occlusion: A Systematic Review and Meta-Analysis
Source: J Ophthalmol. 2022 Dec 28;2022:4823677. doi: 10.1155/2022/4823677 (PMC11390223; doi:10.1155/2022/4823677)
Supplement: Supplementary Materials — Supplementary Material S1: PRISMA 2020 Checklist. Supplementary Material S2: search terms for each database. Supplementary Material S3: a list of excluded studies by reading the full text. Supplementary Material S4: components of Chinese herbal formulas used in the included trials. Supplementary Material S5: table of subgroup analysis. Supplementary Material S6: forest plot of subgroup analysis. Supplementary Material S7: characteristics of adverse events for included studies. Supplementary Material S8: a sensitivity analysis. Supplementary Material S9: forest plot of CMT at 1 month (after removing Hao's study). [file 4823677.f1.zip › Supplementary Material S5.pdf]

Supplementary Material S5. Table of subgroup analysis.

Outcomes	Subgroup	Trial (n)	Sample size (n)	Statistical method	Effect estimate (95% CI)	*p*	I^2
BCVA at 6 months	RVO subtype						
	BRVO	2	162	MD, Random	-0.12 [-0.32, 0.07]	0.22	92%
	CRVO	1	60	MD, Random	-0.02 [-0.23, 0.19]	0.85	/
	RVO	2	207	MD, Random	-0.11 [-0.17, -0.04]	0.0008	0%
	Course of treatment						
	< 12W	2	212	MD, Random	-0.06 [-0.12, 0.01]	0.1	46%
	⩾12W	3	217	MD, Random	-0.15 [-0.25, -0.05]	0.004	51%
CMT at 1 month	RVO subtype						
	BRVO	4	314	MD, Random	-40.90 [-90.72, 8.92]	0.11	93%
	CRVO	2	120	MD, Random	-62.09 [-104.22, -19.95]	0.004	0%
	RVO	2	99	MD, Random	-18.24 [-52.71, 16.24]	0.3	35%
	Types of anti-VEGF agents						
	ranibizumab	4	256	MD, Random	-6.09 [-20.02, 7.84]	0.39	0%
	bevacizumab	1	60	MD, Random	-75.00 [-124.90, -25.10]	0.003	/
	conbercept	2	160	MD, Random	-71.92 [-174.73, 30.88]	0.17	96%
	Not clear	1	57	MD, Random	-40.11 [-86.43, 6.21]	0.09	/
	Course of treatment						
	< 12W	2	104	MD, Random	-0.43 [-16.38, 15.52]	0.96	0
	≥ 12W	6	369	MD, Random	-53.10 [-92.09, -14.10]	0.008	84%

CMT at 6 months	RVO subtype						
	BRVO	3	254	MD, Random	-63.49 [-85.97, -41.01]	< 0.0001	53%
	CRVO	2	120	MD, Random	-111.43 [-212.37, -10.49]	0.03	84%
	RVO	4	309	MD, Random	-41.84 [-79.17, -4.52]	0.03	93%
	Types of anti-VEGF agents						
	ranibizumab	5	316	MD, Random	-58.75 [-92.10, -25.40]	0.0006	88%
	bevacizumab	1	60	MD, Random	-60.00 [-117.69, -2.31]	0.04	/
	conbercept	1	100	MD, Random	-70.76 [-101.03, -40.49]	< 0.0001	/
	Not clear	2	207	MD, Random	-58.25 [-160.00, 43.51]	0.26	98%
	Course of treatment						
	< 12W	3	254	MD, Random	-73.36 [-111.58, -35.14]	0.0002	88%
	≥12W	6	429	MD, Random	-51.48 [-82.84, -20.11]	0.001	88%
