# Supplementary material for: Chinese Herbal Medicine Combined with Intravitreal Antivascular Growth Factor Agents in Treatment of Macular Edema Secondary to Retinal Vein Occlusion: A Systematic Review and Meta-Analysis
Source: J Ophthalmol. 2022 Dec 28;2022:4823677. doi: 10.1155/2022/4823677 (PMC11390223; doi:10.1155/2022/4823677)
Supplement: Supplementary Materials — Supplementary Material S1: PRISMA 2020 Checklist. Supplementary Material S2: search terms for each database. Supplementary Material S3: a list of excluded studies by reading the full text. Supplementary Material S4: components of Chinese herbal formulas used in the included trials. Supplementary Material S5: table of subgroup analysis. Supplementary Material S6: forest plot of subgroup analysis. Supplementary Material S7: characteristics of adverse events for included studies. Supplementary Material S8: a sensitivity analysis. Supplementary Material S9: forest plot of CMT at 1 month (after removing Hao's study). [file 4823677.f1.zip › Supplementary Material S6.pdf]

Supplementary Material S6. Forest plot of subgroup analysis.

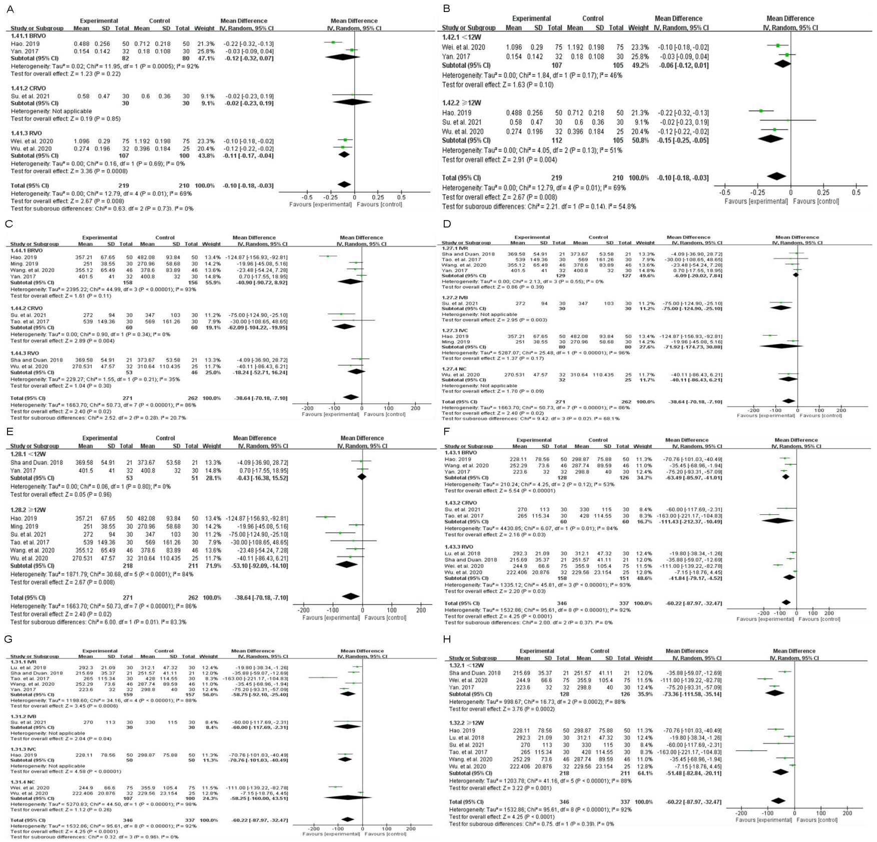

(A) BCVA at 6 months (RVO subtype). (B) BCVA at 6 months (Course of treatment). (C) CMT at 1 month (RVO subtype). (D) CMT at 1 month (Types of anti-VEGF agents). (E) CMT at 1 month (Course of treatment). (F) CMT at 6 months (RVO subtype). (G) CMT at 6 months (Types of anti-VEGF agents). (H) CMT at 6 months (Course of treatment).
