# Supplementary material for: Chinese Herbal Medicine Combined with Intravitreal Antivascular Growth Factor Agents in Treatment of Macular Edema Secondary to Retinal Vein Occlusion: A Systematic Review and Meta-Analysis
Source: J Ophthalmol. 2022 Dec 28;2022:4823677. doi: 10.1155/2022/4823677 (PMC11390223; doi:10.1155/2022/4823677)
Supplement: Supplementary Materials — Supplementary Material S1: PRISMA 2020 Checklist. Supplementary Material S2: search terms for each database. Supplementary Material S3: a list of excluded studies by reading the full text. Supplementary Material S4: components of Chinese herbal formulas used in the included trials. Supplementary Material S5: table of subgroup analysis. Supplementary Material S6: forest plot of subgroup analysis. Supplementary Material S7: characteristics of adverse events for included studies. Supplementary Material S8: a sensitivity analysis. Supplementary Material S9: forest plot of CMT at 1 month (after removing Hao's study). [file 4823677.f1.zip › Supplementary Material S7.pdf]

Supplementary Material S7. Characteristics of adverse events for included studies.

Study ID	Intervention		Course of treatment (weeks)	Adverse Effects	
	T	C		T	C
Hao. 2019	IVC + CHM	IVC	24	NR	NR
Wu, 2020	anti-VEGF + CHM	anti-VEGF	12	One case of increased intraocular pressure	NR
Tao, 2017	IVR +CHM	IVR	12	NR	NR
Yan, 2017	IVR +CHM	IVR	4	Three cases of subconjunctival hemorrhage	Two cases of subconjunctival hemorrhage
Ming, 2019	IVC +CHM	IVC	12	One case of subconjunctival hemorrhage	NR

T, treatment; C, control; NR: Not Reported.
